# Cascaded processing in written compound word production

**DOI:** 10.3389/fnhum.2015.00207

**Published:** 2015-04-21

**Authors:** Raymond Bertram, Finn Egil Tønnessen, Sven Strömqvist, Jukka Hyönä, Pekka Niemi

**Affiliations:** ^1^Department of Psychology, University of TurkuTurku, Finland; ^2^Center for Reading Research and Department of Education, University of StavangerStavanger, Norway; ^3^Department of Linguistics and Phonetics, University of LundLund, Sweden

**Keywords:** morphology, finnish, compound words, writing, cascaded processing, linguistic processing, motor processes, syllable

## Abstract

In this study we investigated the intricate interplay between central linguistic processing and peripheral motor processes during typewriting. Participants had to typewrite two-constituent (noun-noun) Finnish compounds in response to picture presentation while their typing behavior was registered. As dependent measures we used writing onset time to assess what processes were completed *before* writing and inter-key intervals to assess what processes were going on *during* writing. It was found that writing onset time was determined by whole word frequency rather than constituent frequencies, indicating that compound words are retrieved as whole orthographic units before writing is initiated. In addition, we found that the length of the first syllable also affects writing onset time, indicating that the first syllable is fully prepared before writing commences. The inter-key interval results showed that linguistic planning is not fully ready before writing, but cascades into the motor execution phase. More specifically, inter-key intervals were largest at syllable and morpheme boundaries, supporting the view that additional linguistic planning takes place at these boundaries. Bigram and trigram frequency also affected inter-key intervals with shorter intervals corresponding to higher frequencies. This can be explained by stronger memory traces for frequently co-occurring letter sequences in the motor memory for typewriting. These frequency effects were even larger in the second than in the first constituent, indicating that low-level motor memory starts to become more important during the course of writing compound words. We discuss our results in the light of current models of morphological processing and written word production.

## Introduction

The processing architecture underlying word production has for a long time been based on spoken language studies. More recently, the development of experimental on-line writing tools have generated studies that are concerned with written word production (e.g., Delattre et al., 2006; Sahel et al., 2008; Kandel et al., 2012; Baus et al., 2013). These studies typically address a number of questions that are related to the intertwinement of central linguistic processes and more peripheral motor processes. The main question here is to what extent linguistic units are planned before and to what extent during motor execution.

Most studies concern the writing^1^ of monomorphemic words and the evidence suggests that much of the planning is completed before motor execution (e.g., Baus et al., 2013). The current study is concerned with the writing of Finnish two-constituent noun-noun compounds (e.g., *tennismaila* “tennis racket”). Studies in language comprehension (e.g., Fiorentino and Poeppel, 2007) and spoken word production (e.g., Bien et al., 2005) have shown that the initial access of compounds may take place via the constituents, but there are also studies showing that it is mediated via whole-word representations (Janssen et al., 2008, 2014). The first issue is thus to investigate whether in written word production compounds are initially accessed as a whole unit (*tennismaila*) or via their constituent components (*tennis* and *maila*).

The second issue addressed in this study concerns the extent to which linguistic planning takes places during motor execution. Given that compounds are typically longer and linguistically more complex than monomorphemic words, it seems more challenging to have a detailed motor execution plan ready before writing them.

The current study investigates these issues by means of a picture-word elicitation paradigm. The introduction will first discuss studies that have investigated the amount of planning completed before production, followed by a discussion of studies that have investigated the amount of additional planning and processes that take place during writing. Finally, these issues will be linked to the model of written word production proposed by Kandel et al. (2011).

### Linguistic planning before production of written and spoken words

A number of studies has investigated to what extent linguistic planning of monomorphemic words is completed before writing is initiated (e.g., Lambert et al., 2007; Baus et al., 2013; Roux et al., 2013). Typically these studies have investigated the effect a linguistic manipulation exerts on writing onset latency (WOT). Baus et al. (2013) elicited monomorphemic words in Spanish by means of a picture naming paradigm and found that high-frequency words elicited shorter WOTs than low-frequency ones. Roux et al. (2013) manipulated the lexicality of letter strings by employing a French word/pseudoword-copying task and found that WOTs are much shorter for words than for pseudowords. Lambert et al. (2007) found both a lexicality and frequency effect in a French word/pseudoword-copying task. Taken together these results suggest that for monomorphemic words the whole orthographic representation is retrieved before motor execution and that the level of activation is determined by word frequency. Lambert et al. also found that WOTs are independent of the number of syllables for real words, but not for pseudowords. This led them to conclude that the syllabic structure of words is not analyzed in detail before writing, but that for pseudo-words—as a result of a lacking whole-word orthographic representation—letter strings are chunked into syllables. However, it seems that for words at least the first syllable is fully prepared for motor production before writing. This claim is supported by a study in German of Will et al. (2004), who found that WOT is correlated with the length of the first syllable. Longer latencies for longer syllables indicate that all letters have been retrieved and handed over to the motor program before writing commences.

There are no studies of written word production that have investigated the effect of morphological complexity on WOT. That is, no written word production study has addressed the question whether morphologically complex words are initially retrieved via their morphemes, the whole-word form or both. However, a few studies in the other language modality of production, speech, have addressed this question. These studies show mixed results. Bien et al. (2005) investigated whether speech onset latencies were sensitive to constituent and/or compound frequency in a position-response association task. In this task participants first learned to associate each compound with a visually marked position on a computer screen, after which they had to produce the relevant compound in response to the appearance of the position mark. Compounds with high-frequency 1st or 2nd constituents elicited shorter response latencies than compounds with low-frequency 1st or 2nd constituents. The manipulation of the whole-word frequency had little effect on response latencies. Similarly, Koester and Schiller (2008) found that reading aloud Dutch compound words as primes (e.g., *jaszak*, “coat pocket”) speeded the response to a subsequently presented picture of the first constituent (*jas* ‘coat), whereas form-related monomorphemic prime words (e.g., *jasmijn*, “jasmine”) did not. Both studies support a decomposition account, which holds that compounds are initially retrieved via their constituents (see Zwitserlood et al., 2000, 2002 for additional evidence).

However, there are two studies that failed to find constituent effects. Janssen et al. (2008) found that production latencies in a picture naming task eliciting compounds in both Mandarin Chinese and English are a function of whole-word rather than constituent frequencies. Janssen et al. (2014) conducted a large-scale regression study on concatenated English compound words and found the same. In the latter study, a large number of potentially confounding variables was controlled ruling out the possibility that the whole-word frequency effect was a result of methodological differences with other speech production studies. The effect for whole-word frequency thus remained reliable, whereas constituent frequency (or constituent family size) effects could not be found.

Janssen et al.'s (2014) results are not only different from those in other spoken word production studies, but also from those in several word comprehension studies. More specifically, constituent effects are reported by several masked priming (e.g., Duñabeitia et al., 2009), visual lexical decision (e.g., Fiorentino and Poeppel, 2007) and eye movement studies (e.g., Pollatsek et al., 2000), indicating that decomposition is involved in the processing of compound words. When Janssen et al. (2014) extracted lexical decision times from the English Lexicon Project (Balota et al., 2007) for the same compounds as in their production experiment, they found both surface and constituent frequency effects. Janssen et al. concluded that when compounds are explicitly available in the input (as in lexical decision or in the picture-word interference experiments), constituents are actively involved in lexical processing. In contrast, when compounds have to be retrieved from semantic memory without recent exposure, they are retrieved as holistic units. The authors propose that taken together the results support a dual route account, where the activation of the decomposed route depends on the nature of the input representation. The present study investigates whether the results of Janssen et al. (2014) showing holistic compound retrieval extend to written word production or whether compounds are decomposed before retrieval, as found in several other speech production and comprehension studies. In case of decomposition, it is possible that only the first constituent is retrieved before writing commences; in this case only a first constituent frequency effect will be observed in WOT.

### Factors that influence written word production during motor execution

According to Damian (2003), central cognitive processes do not influence spoken word production once motor execution (i.e., articulation) has started. However, as Delattre et al. (2006) have argued, this is clearly not the case for motor execution during writing. According to them, there is more scope for cascaded processing in writing than in speaking, as writing (a) is a less practiced activity than speaking; (b) has evolved much later than speaking and (c) typically takes more time than speaking. Several studies indeed show that linguistic planning in general and morphological planning in particular take place during the motor execution phase of written word production. For example, in a handwriting study of Kandel et al. (2008), the inter-letter interval (ILI) between the root and the suffix in derivational suffixed words (e.g., boulette “small ball”) was compared with the ILI at the same position in pseudosuffixed words (e.g., goélette “caravel”). It was found that ILIs prior to the suffix were longer for suffixed than pseudosuffixed words. This led the authors to conclude that the writing system anticipated the production of the suffix and that letters are grouped in linguistically motivated chunks. Kandel et al. (2012) replicated these findings and also showed that letter durations (the time it takes to write a letter) before morpheme boundaries are inflated in comparison to letter durations before pseudoboundaries. An interesting additional finding in this study was that the results were only obtained for suffixed but not for prefixed words.

A typewriting study of Sahel et al. (2008) investigated by means of a word copying task whether second constituent and/or whole word frequency predicted the inter-key intervals (IKIs) between the first constituent and second constituent of German compound words. They found that IKIs were affected by both and argued that these results lend support to a dual-route account, which postulates that whole-word and decomposition procedures run in parallel and interact with one another. However, given that the study did not consider WOT as a dependent measure, no conclusions about initial compound retrieval can be drawn. Weingarten et al. (2004) found that two-letter sequences (bigrams) at morpheme boundaries in German compounds elicited much longer IKIs than bigrams at pure syllable boundaries or intrasyllabic bigrams transitions. Thus, in a word like *Maiskolben* (“corncob”), the IKI at the morpheme boundary between *s* and *k* is much longer than the IKI at the pure syllable boundary between *l* and *b* or the IKIs of all other intrasyllabic bigrams. In other words, a constituent boundary prolongs the writing of two adjacent letters, much more than any other factor. This is even the case when exactly the same bigrams are considered at different positions within words (intrasyllabic, syllable boundary, constituent boundary, see Weingarten et al., 2004). Taken together, the results imply that at least for suffixed and two-constituent compound words the second constituent morpheme is activated (or reactivated) at the morpheme boundary.

Weingarten et al. (2004) also report syllable-based effects during writing; the IKIs for the intersyllabic bigrams in their study were much longer than intrasyllabic bigrams. These syllable-based effects are also reported in Spanish and French (Kandel and Valdois, 2006; Kandel et al., 2006; Álvarez et al., 2009). Moreover, they are found with different inputs (visual words, auditory words, pictures), in different dependent measures (IKIs, ILIs, letter writing duration, gaze lifts) and with different populations (adults, children, bilinguals). In all these cases it is reported that writing slows down at or around the syllable boundary, indicating that the system prepares the production of upcoming syllables whilst writing. Kandel et al. (2006) note that the role of syllables is likely to be more prominent in languages with clear syllable structure. Finnish—the language of current investigation—has a regular syllabic structure with clearly defined syllable boundaries, no ambisyllabicity and stress falling practically always on the first syllable. Thus, we may expect solid syllable effects for Finnish as well.

Apart from the impact of clear linguistic boundaries, certain letter combinations within or across such boundaries also may affect motor execution during writing. Weingarten et al. (2004) report that gemination, the doubling of vowels or consonants, leads to faster typing of the second letter in comparison to the second letter of letter sequences with different letters. This has an obvious explanation: the finger is already positioned on the target key when typing the second letter. This benefit is not self-evident in handwriting, where similar movements have to be made for writing the first letter and the second letter in geminate pairs. However, Kandel et al. (2014) reported shorter letter production times also for the second letter in a geminate pair (the second *s* in *Lisser* compared to the *t* in *Lister*) in handwriting. Moreover, they did not find the typical inflation effect for ILIs at syllable boundaries for words with gemination (*Lisser*). The available evidence thus suggests that also in handwriting there is some kind of motor preparation effect that speeds up the production of the second letter in the geminate pair. Interestingly, Kandel et al. (2014) found that this kind of motor preparation takes place at the expense of writing the initial letters of a word. More specifically, they found longer writing durations for the first three letters (*Lis*) in the geminate word (*Lisser*) than in the control word (*Lister*). This implies that gemination requires additional planning during motor execution which slows down the writing of the initial letters. All in all, the results led Kandel et al. (2014) to conclude that gemination annuls the syllable-by-syllable programming strategy.

Kandel et al. (2011) investigated the interaction between bigram frequency and syllable boundary in handwriting. For visual word recognition it has been argued that readers become sensitive to orthographic regularities like the co-occurrence of adjacent letters (bigrams, trigrams), such that frequently co-occurring letters develop stronger links and can be processed more quickly than less frequent sequences (Seidenberg, 1987; Treiman and Zukowski, 1988). As intrasyllabic letters co-occur more often than intersyllabic letters (Adams, 1981), the syllable effects reported in comprehension (e.g., Prinzmetal et al., 1986) and production studies (e.g., Kandel et al., 2006) may be bigram frequency effects in disguise. Doignon and Zagar (2005) showed that this is partly the case, as their syllable effects were attenuated for high-frequency bigrams at the syllable boundary. However, the fact that the syllable effect was not completely wiped out by high-frequency bigrams indicates that the syllable is a functional processing unit during visual word comprehension (see also Rapp, 1992). Similarly, Kandel et al. (2011) found that a relatively frequent bigram at the syllable boundary increases ILIs for children and adults alike, but not as much as would be expected on the basis of bigram frequency alone. That is, a high-frequency bigram at a syllable boundary is not written as fast as the same high-frequency bigram in intrasyllabic position.

### A model for written word production

To account for the findings presented above, Kandel et al. (2011) proposed a model of written word production that includes linguistic modules, a spelling module and motor modules (see **Figure 2**). The linguistic modules pertain to the activation of intentions and gearing up the semantic and syntactic system including semantic retrieval. The spelling module includes a number of abstract processing levels that are active in parallel. In the initial phase of the spelling module, the orthographic representation of the whole word is retrieved. This representation activates in turn syllables at the syllable level, which in turn activate letters at the letter level. The letter level also stores knowledge about letter co-occurrence (bigrams, trigrams) as well as knowledge about phoneme-grapheme correspondences. Subsequently letters will be transferred to the motor modules, where graphomotor planning for handwriting takes place, including the selection of allographs (e.g., uppercase or lower case), leading to the eventual production of letters. Note that this phase is different for typewriting, as for typewriting a series of hand and finger movements have to be programmed in standard keyboard space (for a more detailed description of written word production models, see Weingarten et al., 2004; Kandel et al., 2011; Purcell et al., 2011). Kandel et al.'s model is derived from the classic Van Galen (1991) model, but differs from it by adding a syllable level and an abstract letter level to the spelling module to account for the syllable and bigram/trigram effects found in several studies.

## Experiment

The current study investigated a number of issues. First, we asked whether retrieval of Finnish compound words (e.g., *tennismaila* “tennis racket”) takes place via morphological constituents (*tennis* and *maila*) or whether retrieval is holistic in nature. To that end, compounds with varying constituent and whole-word frequencies were selected and these frequency variables were entered in the regression analyses as predictors for Writing Onset Time (WOT). It was assumed that if retrieval took place holistically, the whole-word frequency would predict WOTs, whereas decompositional retrieval would be predicted by constituent frequency effects. In order to investigate in more detail what is prepared *before* writing we entered a number of other variables as well. We anticipated that at least the length of the 1st syllable would affect WOT (cf. Weingarten et al., 2004).

In order to investigate how much linguistic planning goes on *during* writing, we extracted all the Inter-Key Intervals (IKIs) between subsequent letters and entered a number of variables as predictors in the regression analyses. In particular, we were interested to investigate to what extent certain linguistic transitions and bigram and trigram frequencies affected IKIs. In our compounds (e.g., *tennismaila* “tennis racket”), we distinguished four different types of transition: intrasyllabic no-boundary transitions (in our example *te, en, ni, is, ma, ai, la*), syllabic gemination transitions (in our example *nn*), pure syllabic transitions (in our example *il*) and morphosyllabic transitions (in our example *sm*). The impact of bigram frequency and syllable boundaries on IKIs was assessed in more detail in an additional IKI-analysis to examine whether effects depended on IKIs appearing in the first or second constituent. More specifically, in this way we assessed the time course of effects within words. Finally, we were interested in whether any of the effects were affected by participants' typing skills, so average typing speed was also added to both analyses. All variables will be described in more detail in the method section.

### Method

#### Participants

Eighteen undergraduate students of the University of Turku participated in the experiment. All were native speakers of Finnish, and had normal or corrected-to-normal vision.

#### Apparatus

The program used for our experiment is called ScriptLog, invented by Strömqvist and Malmsten (1998) and further developed by Strömqvist et al. (2006). Scriptlog is a program with two windows, an elicitation window and an editor window. In the editor window the participant types the word that corresponds to the picture presented in the picture window. The program registers the production time of letters and words, typing errors and their corrections, inter-key intervals and writing onset time (among other things). In other words, it allows for the extraction of a multitude of measures that give a detailed insight into the writing process.

#### Materials

Before the experiment proper, we conducted a paper-and-pencil pretest to assure that the pictures would elicit the intended compounds. In this test 15 native Finnish students wrote down the name of 50 preselected target pictures that supposedly would elicit compound words. They also rated the pictures' visual complexity (from 1, visually simple, to 5, visually complex) and typicality (how well does the picture correspond to your own mental representation of this item/object; from 1, not at all, to 5, perfect match). For the experiment proper, only those pictures were included that elicited at least 73.3 of the time the intended compound (average 94.4%) and had an average typicality rating of at least 3. These criteria allowed us to select 26 target pictures that elicited noun-noun compounds. The lexical statistics of these compounds were extracted from an unpublished computerized newspaper corpus of 22.7 million word forms, assessed with the help of the WordMill database program of Laine and Virtanen (1999). The 26 target compounds are listed in Supplementary Material. Table 1 lists the average and the range of the ratings and variables that were included in the analyses. The experimental items were mixed with 26 filler items. These filler items were pictures that were intended to elicit monomorphemic words (e.g., lasi “glass,” vasara “hammer,” kana “chicken”).

**Table 1:** **Properties of the target compounds and the participants (Typing Speed)**.

**Variable**	**Average**	**Range**
1st-constituent frequency^a^	38.81	0.5–233.3
2nd-constituent frequency^a^	62.11	0.1–378.1
Mean lemma frequency^a^	4.8	0.1–32.2
Word length^b^	11.0	8–13
1st constituent length^b^	5.8	4–8
2nd constituent length^b^	5.3	4–8
1st syllable length^b^	2.8	2–4
2nd syllable length^b^	2.3	2–4
Bigram frequency^c^	6.82	3.37–10.78
Initial trigram frequency^c^	0.65	0.07–2.70
Final trigram frequency^c^	0.94	0.05–4.06
Naming score	0.94	0.73–1.00
Visual complexity rating^d^	2.70	1.27–4.18
Typicality rating^d^	4.15	3.33–4.87
Typing speed in ms	2443	1283–3746

a *All values scaled to one million*.

b *Length in characters*.

c *Scaled to one thousand*.

d *Rating scale from 1 to 5*.

#### Procedure

**Figure 1:**
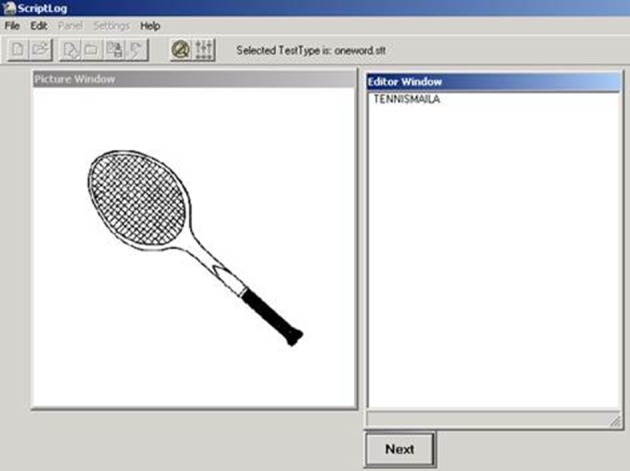
**The two windows of the elicitation tool ScriptLog**. Upon presentation of the picture in the left window, the participant types the picture name in the right window. In this example the participant writes the word *tennismaila* “tennis racket.” Going with the mouse cursor to “next” and pressing a mouse button will make the next picture appear.

After instruction, participants were exposed to 52 pictures in the picture window (see Figure 1), the first four pictures being filler items eliciting monomorphemic words. After that, the pictures eliciting monomorphemic filler words and those eliciting target compound words were presented in random order, but such that no more than 3 compound items appeared after each other. The task was to write down what each picture represented. The participants started the experiment by pointing the mouse cursor to the “start”-button on the screen and clicking the left mouse button. After that, a picture appeared in the left window of the screen. In the right window, the editor window, the participant had to write down as quickly and accurately as possible what the picture represented. After this the mouse cursor had to be pointed to the “next”-button on the screen and the left mouse button had to be clicked again. This made the following picture appear and the same procedure was repeated until all 52 pictures were responded to. The experiment lasted approximately 10–15 min.

#### Dependent variables and predictors

We used two written word production measures as the dependent variables in our analyses. The first one was writing onset time (*WOT*), the time between picture presentation and the first keystroke; in addition, we considered the inter-key intervals (*IKI*), the time in between each keystroke. The independent variables included in all statistical models are listed in Table 1 and include typicality (*Typical*, 1–5), visual complexity (*VisCom*, 1–5), number of syllables (4–5), log lemma frequency (*LLemfreq*), log 1st and 2nd constituent frequency (*LFreq1c, LFreqc2*), log bigram frequency (*LBiFreq*; *for WOT average bigram frequency and for IKI individual bigram frequencies were entered in the model*), log frequency of the initial (*LIni3*); and final trigram (*LFin3*), whole word and 1st and 2nd constituent length (*LenWW, Len1c, Len2c*), 1st and 2nd syllable length (*LenSyl1, LenSyl2*) and typing proficiency (*TypingSpeed*). For the latter variable, average writing time of the compounds was used as an approximation of typing proficiency. For *IKI* the type of linguistic transition, *LingTrans*, between two letters was still added to the analyses. This factor included four levels: no boundary *n* (*N* = 164), syllabic boundary *s* (*N* = 44), morphosyllabic boundary *m* (*N* = 26), and geminate *g* (*N* = 27). Finally, we reanalyzed the data for *IKI* (*IKI_2*) to assess the time course of effects within words by including constituent as a factor (*Const*, 1–2). For these analyses, the morphosyllabic condition had to be excluded, as the boundary for this condition is exactly between the first and second constituent; we also excluded the geminate condition in order to obtain a purer comparison between the syllable boundary and no-boundary conditions. For all the models, we included participants and items as random effects; other variables did not improve the random effect structure. Variables with a high mutual correlation were decorrelated before entering into the statistical models (e.g., Len2c and LFreqc2 were highly correlated, so we used residualized *Len2c, Len2c* from which the influence of *LFreqc2* was partialled out). The fixed effects of the dependent measures are listed in the Supplementary Material.

### Results

The data were analyzed using linear mixed effects models with participants and items as crossed random effects, while making use of the lme4 package (Bates et al., 2013) for R statistical software (R Core Team, 2013). Separate models were fitted for both dependent measures. The measures were log-transformed in order to normalize their distributions. Trials in which the target word was misspelled, initially mistyped or not the intended compound were excluded before analyses (16% of trials). Values that were 3 SDs smaller or larger than the grand mean were excluded (1.5% of the trials for WOT and 1% of the trials for IKI). No further data trimming was done before analyses. We report models with the effects that retained statistical significance in the stepwise backward elimination procedure. More precisely, we first included all the predictors and subsequently removed the least predictive predictor in each round until we ended up with a model with only significant predictors, |*t*| > 1.96. We also made sure by model comparison that each predictor significantly improved the explanatory power of the model. The model specifications are presented in detail in Supplementary Material.

#### WOT

There was a significant effect for *LLemFreq*, |*t*| > 2. The more frequent the word, the more quickly participants started to type. In addition, the effect for *Typical* was significant, |*t*| > 2. The more clearly a picture corresponded to participants' own mental representation of a given object, the shorter the *WOT*. Significant effects were also found for *TypingSpeed* and *Syl1Len*, both |*t*|*s* > 2. Faster typists initiated typing earlier than slower ones and longer first syllables elicited longer WOTs than short first syllables. Other variables did not make significant contributions to the model. For instance, neither the effects of *LFreq1c* and *LFreqc2* (|*t*|s < 1.3, when entered alone) nor any interaction came close to significance^2^.

#### IKI

There was a clear effect for *LingTrans*, with different *IKIs* for all four types of transitions, all *t*s > 2. The time between keystrokes was smallest in the case of geminates; it was significantly longer when there was no boundary, still longer at the syllable boundary and longest when there was a morphosyllabic boundary (*g* = 155 ms; *n* = 217 ms; *s* = 274 ms; *m* = 377 ms). The two other variables that affected *IKI*s were *LBiFreq, t* > 2, and *LFin3, t* > 2, with words including more frequent bigrams and more frequent final trigrams generating shorter *IKIs* than words with less frequent ones. The best model though included interactions between *LingTrans* and *LBiFreq*, with interactions between the following levels: *m* X *s, m* X *g, n* X *s*, and *n* X *g*. The interactions reflected that the effect for *LBiFreq* was larger for *IKI*s at morphemic boundaries and no boundaries than at pure syllable boundaries or for geminates. Separate analyses revealed that the effect for *LBiFreq* was significant for all transitions apart from gemination.

#### IKI2

In order to assess the time course of effects within words, we reanalyzed the IKI-data by including constituent (*Const*, 1 or 2) as a factor, but for the no boundary and syllable boundary conditions only. For this measure there were clear main effects for *LingTrans, LBiFreq, LFin3*, and *Const*, all *t*s > 2. For the first three variables the effects were the same as in the initial *IKI*-analysis. The effect of *Const* indicated that *IKIs* were shorter in the second constituent than in the first constituent. The best model though included interactions between *LingTrans* and *LBiFreq* and between *Const* and *LBiFreq*, both *t*s > 2. The first interaction indicated again that the effect of *LBifreq* was larger for *IKIs* at intrasyllabic positions than at syllable boundaries. The second interaction indicated that the effect of *LBifreq* was larger for *IKIs* during second constituent writing than during first constituent writing. We further explored the latter interaction, by separately analyzing the first and second constituent of the syllable boundary and the no boundary condition. These analyses showed that *LBifreq* did not affect first constituent IKIs at syllable boundaries, *t* < 1, but had a significant effect on second constituent IKIs at syllables boundaries, *t* > 2. For the no-boundary IKIs the *LBifreq* effect was significant for both constituents, both *t*s > 2, be it that it was larger for the second constituent.

## Discussion

The current study set out to investigate whether Finnish compounds are retrieved holistically or via constituents, while at the same time it investigated what linguistic planning takes place before and during motor execution when typewriting these compounds. To assess linguistic planning before writing, we used WOT as the dependent measure; for processes during writing, we opted for the IKI between the typing of two subsequent letters.

It was found that whole-word frequency rather than constituent frequency was a solid predictor for WOT, indicating that initial retrieval is holistic in nature. Moreover, it was found that picture typicality, typing speed and the length of the first syllable had an impact on WOT. The picture typicality effect indicates that less prototypical pictures require more processing resources to retrieve the correct semantic concept. The typing speed effect indicates that more skillful typists manage to activate their motor program more quickly than less skillful ones. Perhaps it also reflects that more skillful typists are faster in placing their right hand back to the keyboard keys after having clicked the mouse to start a new trial.

With respect to the first syllable length effect, it can be argued that if only the first phoneme would have been prepared before writing, the length of the 1st syllable should not have mattered. Given that longer first syllables led to longer WOTs, it has to be concluded that the first syllable is fully prepared before writing is initiated, including the retrieval and activation of the motor program for all first syllable graphemes (see Weingarten et al., 2004, for a similar argumentation).

The IKI-results indicated that linguistic planning is not fully ready before writing, as linguistic boundaries clearly caused a delay during writing. More specifically, IKIs were longer for letter sequences around a syllable and morphosyllabic boundary than for intrasyllabic sequences. The IKIs were shortest for geminates, whereas morphosyllabic boundaries generated the longest IKIs. It thus seems that linguistic planning cascades into the actual motor execution phase and linguistic units need to be retrieved or reactivated whilst writing. Interestingly, bigram and trigram frequency also affected IKIs, even more so in the second part of the compound word than in the first part. Higher bigram frequencies led to shorter IKIs for intrasyllabic, syllabic and morphosyllabic letter sequences, but the bigram effect did not appear at syllable boundaries in the first constituent and was smaller for intrasyllabic sequences in the first than in the second constituent. Moreover, whereas the frequency of the initial trigram, always appearing in the first constituent, did not affect IKIs; higher frequencies of the final trigram—always located in the second constituent—clearly led to shorter IKIs.

### Retrieval of the orthographic representation

The picture-word written production task requires object identification and retrieving the semantic concept, after which an orthographic representation from the orthographic long-term memory store (O-LTM) can be retrieved (see Purcell et al., 2011). This retrieval process can take different shapes in case noun-noun compound words are involved, as these compounds contain two words which have their own orthographic representations. Typically, the constituent words are more frequent than the compound word and are therefore likely candidates to be activated before the whole compound word. Several compound word studies in spoken language production suggest that constituents are involved at an early stage in word retrieval. For example, Bien et al. (2005) showed by a position-response association task that response latencies where predicted by constituent frequencies rather than whole-word frequency. Several picture-word interference studies showed priming of constituents (*jas* ‘coat) by earlier presented compound words (e.g., *jaszak*, “coat pocket”), but not by orthographic controls (e.g., jasmijn, “jasmine”; Zwitserlood et al., 2000, 2002; Koester and Schiller, 2008). In addition, in reading comprehension constituent frequency effects are omnipresent in masked priming (e.g.,Duñabeitia et al., 2009), visual lexical decision (e.g., Fiorentino and Poeppel, 2007) and eye movement studies (e.g., Pollatsek et al., 2000; White et al., 2008). All these studies thus show that constituents are involved in initial access/retrieval of compounds.

However, two studies in speech production on compounds using the picture naming task did not find any constituent effects (Janssen et al., 2008, 2014). On the contrary, these studies found that production latencies were predicted by whole-word frequency in both Mandarin Chinese and English. Using an equivalent to this task in written word production, we find exactly the same results as Janssen and associates. Thus, we also conclude that initial retrieval of compounds in production is holistic in nature. However, it may well be the case that this retrieval procedure is not written in stone. Janssen et al. argue that for all studies where constituent effects are found the compounds were visually presented. In the position-response association task of Bien et al. (2005), participants were exposed to compounds several times in the training phase, during which they learned to link a specific compound with a specific position. Similarly, in the picture interference paradigm compounds are first visually presented, before they are being produced (Zwitserlood et al., 2000, 2002; Koester and Schiller, 2008). In these paradigms one does actually not know whether the constituent effects are solely on the production side, or whether the initial visual presentations or perhaps the earlier production of the compound has triggered decompositional access and retrieval. In that sense one may say that a basic picture naming paradigm in which the compound words are not explicitly presented beforehand is a purer task to assess how compounds are produced. It seems that under these context-free circumstances holistic retrieval is the most likely procedure, in both spoken and written word production. However, we do agree with Janssen et al.'S (2014) conclusion that their results together with the results of other compound studies where constituent effects are found suggest a dual route system. That is, we also would argue that both processing routes are at work during compound retrieval, whereby under context-free circumstances (picture naming) the whole-word route is the faster one to deliver. However, as soon as constituents receive some prior stimulation (picture interference, cueing paradigms) the decomposition route is boosted and will be involved in initial compound retrieval. We therefore predict that when using in written word production for instance a (compound) word-copying paradigm (presenting the compound words instead of pictures in the elicitation window), onset latencies will be predicted by constituent frequencies as well. We leave it to further research to test this hypothesis.

### Cascaded processing during written word production

As in previous studies, we also found that during motor execution intervals between keystrokes are neither equal nor random, but dictated by a number of linguistic properties within the compound. The effects of gemination mimic the results of Weingarten et al. (2004) reflecting that it is fairly easy to strike the same button twice on a keyboard, once the typist has sorted out that the word contains a double vowel or consonant at a certain position. However, the inflated IKIs at syllabic and morphosyllabic boundaries as well as the impact of bigrams and trigrams—most prominent in the second part of the compound—cannot be ascribed to the keyboard configuration.

**Figure 2:**
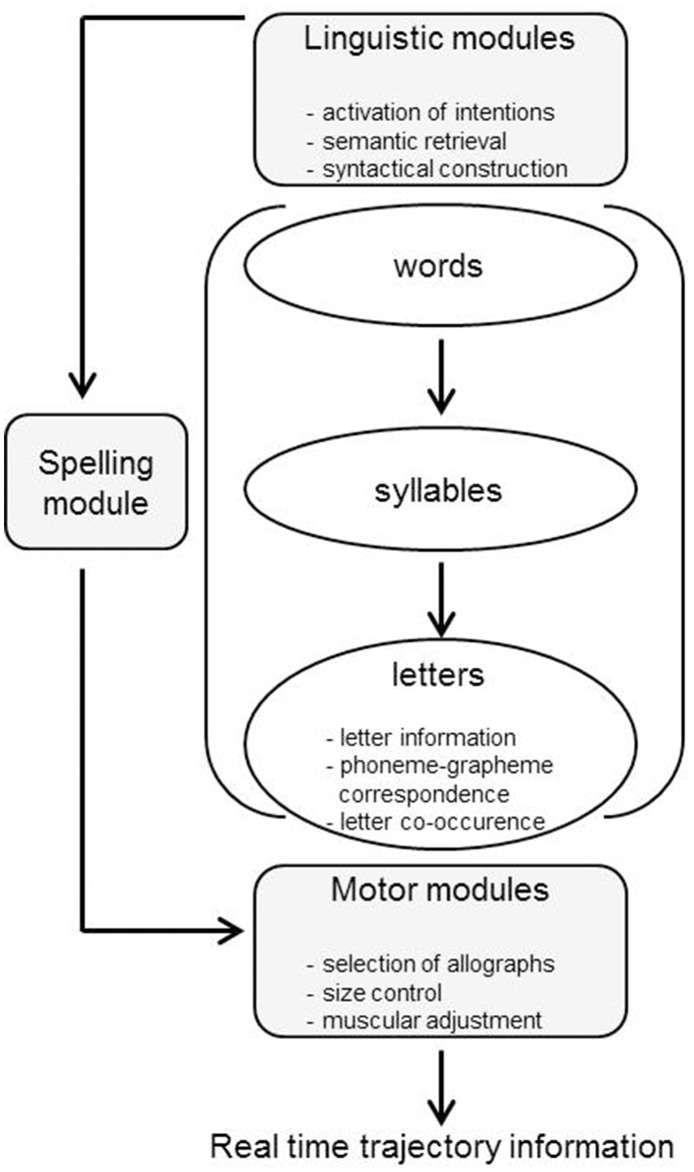
**Handwriting model for written word production adapted from Kandel et al. (2011)**.

Kandel et al. (2011) proposed that the model of Van Galen (1991) should be extended with a syllable level, as there is ample evidence that the syllable is a functional processing unit during written word production, at least in languages with clear syllable structure (see Figure 2). The syllable effect (longer intervals for letter sequences at syllable boundaries than intrasyllabic letter sequences) that we found in Finnish adds to this body of evidence. Kandel et al. (2011) describe how a bisyllabic word like VILAIN is produced in handwriting. After activating the linguistic modules and the orthographic representation of the whole word, the syllable module is activated which informs the writing system about the syllabic structure of the word (VI + LAIN). The first syllable (VI) is then fed forward via the letter module to the motor module for production, while at the same time the next syllable (LAIN) is “activated on-line” (p. 1320). We presume that this implies that both syllables are fed forward to the letter level and that only the first syllable (VI) is then handed over to the motor modules. In a subsequent phase—while the first syllable is being produced—the next syllable (LAIN) is handed over to the motor modules. It also has to be assumed that handing over of the second syllable to the motor modules is not fully completed during the production of the first syllable, but that it spills over to some extent to the syllable boundary, hence the inflated inter-key intervals at this boundary.

The next question is at what level the bigram frequencies come into play. Kandel et al. (2011) suggest that letter co-occurrence information is stored at the letter level. This would mean that high frequency bigrams more quickly reach activation threshold at this level and are handed over to the motor modules than low-frequency bigrams, probably by virtue of stronger activation links between the two letters in the bigram. For intrasyllabic bigrams this procedure seems very plausible, but one may ask—if syllables are handed over to the motor modules one at a time—how the frequency of intrasyllabic bigrams can modulate IKIs. We think that it is likely that these effects also partly reside in the motor modules; that is, it is likely that procedural (finger) muscle memory is involved here with more automatized behavior in case of frequently co-occurring letter sequences than more rarely co-occurring sequences. To put it simply, the fingers are more used to type sequences of letters that frequently co-occur and typing such sequences is more automatized than typing infrequently occurring sequences. Higher bigram frequencies are gluing linguistic units like syllables together—even though their motoric encoding is sequential—by more quickly handing over a syllable to the motor modules. However, it should be noted that this only happens in the second constituent and bigram frequency effects are still larger for intrasyllabic than intersyllabic bigrams. Thus, it has to be concluded that procedural motor memory does not completely wipe out linguistically motivated processing (in this case syllable-based processing).

One may also wonder why bigram and trigram effects are stronger toward the end of the word than in the beginning. As noted above, bigram frequency actually does not affect IKIs in the first but only in the second constituent at syllable boundaries. Moreover, also the effect for intrasyllabic bigrams is stronger in the first than in the second constituent and even trigram frequency only exerts an effect in the second constituent. We think that this reflects that the motor program needs some warming-up during the typing of a long compound word. That is, initially typing is more linguistically motivated (hence the lack of a bigram frequency effect at the first syllable boundary), but upon arrival to the second constituent, low-level automatisms start guiding the processing.

A subsequent question that needs to be asked is to what extent morphological encoding takes place during written compound word production. The longer IKIs at the morphosyllabic boundaries in comparison to the pure syllable boundaries suggest that there is at least some morphological influence during writing. This is confirmed by similar findings of Weingarten et al. (2004) in German and by Kandel et al. (2012) in French. However, it is unclear whether the boundary effect implies (late) activation of the first constituent at the constituent boundary, whether it indicates that the second constituent is retrieved at the boundary, or both. At least Kandel et al. (2012) suggest that their handwriting model should be still further extended with a morphemic level located between the word and the syllable level. However, if syllable effects are observed before morpheme effects, as observed in our study as well as by Kandel et al. (2012), one may wonder whether the morphemic level should be above the syllable level. In addition, in case the morpheme boundary does not coincide with the syllable boundary, as in the Kandel et al. (2012) study (e.g., pruneau, syllabified as pru.neau, with morphological structure prun/eau), the question is how syllable structure is going to be recovered after it is first violated by dividing the word in morphemes. In sum, one can say that morphological structure has an impact on on-line written word production (see also Sahel et al., 2008), but the current empirical evidence does not allow to make conclusions about how morphology should be incorporated in a model of written word production.

Finally, two additional points have to be made. First, it needs to be noted that the phonological level is not included in current models of written word production. Yet, it is undoubtedly the case that during retrieval phonological representations get activated as well as that phonological rehearsal will take place during the writing process. Second, even though we have argued for a cascaded processing architecture, it is likely that the processing system is to some extent interactive as well. For one thing, since morphemes can be subsyllabic, syllabic, and multisyllabic, it is likely that we need an interactive model to capture the reality of the processes going on during writing. We leave it to further studies to address these issues in more detail.

### Concluding remarks

The current study showed that typewriting is an intricate interplay between central linguistic processing and peripheral motor processes. Compound words seem to be retrieved as whole orthographic units and the first syllable is fully prepared before writing commences. However, linguistic planning is not fully ready before writing, but cascades into the motor execution phase where additional planning is needed. In terms of the model by Kandel et al. (2011), one could say that graphemes beyond the first syllable are handed over to the motor system only during or after the production of the first syllable. In addition, we showed that letter co-occurrence also plays a role in written word production, suggesting the involvement of automatized routines of motor memory.

## Author contributions

RB, JH, and PN were involved in conducting and analysing the experiment as well as in designing and writing the study; FT and SS were involved in designing and writing the study. SS also developed the program Scriptlog by which the study was conducted. All authors approved the final version of the current submission. We thank the two reviewers for their helpful suggestions.

### Conflict of interest statement

The authors declare that the research was conducted in the absence of any commercial or financial relationships that could be construed as a potential conflict of interest.

## Footnotes

^1^Note that with writing we refer to both handwriting and typewriting.

^2^Initially, the compounds were selected in such a way that there were 2 factorial manipulations included. The first manipulation concerned whole-word frequency, with 10 high-frequency compounds (on average 9 per million) and 10 low-frequency compounds (on average 1 per million). The second manipulation concerned 1st constituent frequency with 10 compounds having a high-frequency 1st constituent (79 per million) and 10 having a low-frequency one (on average 6 per million). For both manipulations other factors (frequencies, lengths) were matched. Similar to the regression analyses, the ANOVAs showed that the effect of whole-word frequency was highly significant (hf: 1406 ms vs. lf: 1723 ms, both *ps* < 0.05), whereas the effect of 1st constituent frequency did not even approach significance (hf: 1588 ms vs. lf: 1600 ms, both *ts* < 1).

## References

[B1] Adams M. J. (1981). “What good is orthographic redundancy?” in *Perception of Print: Reading Research in Experimental Psychology*, eds Tzeng O. J. L. Singer H. (Hillsdale, NJ: Lawrence Erlbaum Associates Inc.), 197–221.

[B2] Álvarez C. J. Cottrell D. Afonso O. (2009). Writing dictated words and picture names: syllabic boundaries affect execution in Spanish. Appl. Psychol. 30, 205–223 10.1017/S0142716409090092

[B3] Balota D. Yap M. Hutchison K. Cortese M. Kessler B. Loftis B.. (2007). The English Lexicon Project. Behav. Res. Methods 39, 445–459. 10.3758/BF03193014 17958156

[B4] Bates D. Maechler M. Bolker B. Walker S. (2013). lme4: Linear Mixed-Effectsmodels using Eigen and S4 (R package version 1.0-5). Avaliable online at: http://CRAN.R-project.org/package=lme4

[B5] Baus C. Strijkers K. Costa A. (2013). When does word frequency influence written production. Front. Psychol. 4:963. 10.3389/fpsyg.2013.00963 24399980 PMC3870946

[B6] Bien H. Levelt W. J. M. Baayen H. R. (2005). Frequency effects in compound production. Proc. Natl. Acad. Sci. U.S.A. 102, 17876–17881. 10.1073/pnas.0508431102 16301521 PMC1287486

[B7] Damian M. (2003). Articulatory duration in single-word speech production. J. Exper. Psychol. Learn. Mem. Cogn. 29, 416–433. 10.1037/0278-7393.29.3.416 12776752

[B8] Delattre M. Bonin P. Barry C. (2006). Written spelling to dictation: sound-to-spelling regularity affects both writing latencies and durations. J. Exper. Psychol. Learn. Mem. Cogn. 32, 1330–1340. 10.1037/0278-7393.32.6.1330 17087587

[B9] Doignon N. Zagar D. (2005). Illusory conjunctions in French: The nature of sublexical units in visual word recognition. Langu. Cogn. Proc. 20, 443–464 10.1080/01690960444000269

[B10] Duñabeitia J. A. Laka I. Perea M. Carreiras M. (2009). Is Milkman a superhero like Batman? Constituent morphological priming in compound words. Eur. J. Cogn. Psychol. 21, 615–640 10.1080/09541440802079835

[B11] Fiorentino R. Poeppel D. (2007). Compound words and structure in the lexicon. Langu. Cogn. Processes 22, 1–48 10.1080/01690960701190215

[B12] Janssen N. Bi Y. Caramazza A. (2008). A tale of two frequencies: Determining the speed of lexical access for Mandarin Chinese and English compounds. Langu. Cogn. Processes 23, 1191–1223 10.1080/01690960802250900

[B13] Janssen N. Pajtas P. E. Caramazza A. (2014). Task influences on the production and comprehension of compound words. Mem. Cogn. 42, 780–793. 10.3758/s13421-014-0396-z 24691582

[B14] Kandel S. Alvarez C. Vallée N. (2008). “Morphemes also serve as processing units in handwriting production,” in *Neuropsychology and Cognition of Language Behavioral, Neuropsychological and Neuroimaging Studies of Spoken and Written Language*, ed M. Baciu (Thiruvananthapuram: Research Signpost, 87–100.

[B15] Kandel S. Álvarez C. Vallée N. (2006). Syllables as processing units in handwriting production. J. Exper. Psychol. Hum. Percept. Perform. 32, 18–31. 10.1037/0096-1523.32.1.18 16478323

[B16] Kandel S. Peereman R. Ghimenton A. (2014). How do we code the letters of a word when we have to write it? Investigating double letter representation in French. Acta Psychol. 148, 56–62. 10.1016/j.actpsy.2014.01.002 24486807

[B17] Kandel S. Peereman R. Grosjacques G. Fayol M. (2011). For a psycholinguistic model of handwriting production: testing the syllable-bigram controversy. J. Exper. Psychol. Hum. Percept. Perform. 37, 1310–1322. 10.1037/0096-1523.32.1.18 21500939

[B18] Kandel S. Spinelli E. Tremblay A. Guerassimovitch H. Alvarez C. J. (2012). Processing prefixes and suffixes in handwriting production. Acta Psychol. 140, 187–195. 10.1016/j.actpsy.2012.04.005 22664316

[B19] Kandel S. Valdois S. (2006). French and Spanish-speaking children use different visual and motor units during spelling acquisition. Langu. Cogn. Processes 21, 531–561 10.1080/01690960500095946

[B20] Koester D. Schiller N. O. (2008). Morphological priming in overt language production: electrophysiological evidence from Dutch. Neuroimage 42, 1622–1630. 10.1016/j.neuroimage.2008.06.043 18674626

[B21] Laine M. Virtanen P. (1999). WordMill Lexical Search Program. Turku: Center for Cognitive Neuroscience; University of Turku.

[B22] Lambert E. Kandel S. Fayol M. Esperet E. (2007). The effect of the number of syllables when writing poly-syllabic words. Reading Writing Interdiscip. J. 21, 859–883 10.1007/s11145-007-9095-5

[B23] Pollatsek A. Hyön,ä J. Bertram R. (2000). The role of morphological constituents in reading finnish compounds. J. Exper. Psychol. Hum. Percept. Perform. 26, 820–833. 10.1037/0096-1523.26.2.820 10811178

[B24] Prinzmetal W. Treiman R. Rho S. H. (1986). How to see a reading unit. J. Mem. Langu. 25, 461–475 10.1016/0749-596X(86)90038-0

[B25] Purcell J. J. Turkeltaub P. E. Eden G. F. Rapp B. (2011). Examining the central and peripheral processes of written word production. Front. Psychol. 2:239. 10.3389/fpsyg.2011.00239 22013427 PMC3190188

[B25a] R Core Team (2013). R: A Language and Environment for Statistical Computing. Vienna: R Foundation for Statistical Computing Available online at: http://www.R-project.org/

[B26] Rapp B. (1992). The nature of sublexical orthographic organization: the bigram trough hypothesis examined. J. Mem. Langu. 31, 33–53 10.1016/0749-596X(92)90004-H

[B27] Roux J. -S. McKeeff T. J. Grosjacques G. Afonso O. Kandel S. (2013). The interaction between central and peripheral processes in handwriting production. Cognition 127, 235–241. 10.1016/j.cognition.2012.12.009 23454797

[B28] Sahel S. Nottbusch G. Grimm H. Weingarten R. (2008). Written production of German compounds: effects of lexical frequency and semantic transparency. Written Langu. Lit. 11, 211–227 10.1075/wll.11.2.06sah

[B29] Seidenberg M. (1987). “Sublexical structures in visual word recognition: access units or orthographic redundancy?” in *Attention and Performance XII: The Psychology of Reading*, ed Coltheart M. (Hillsdale, NJ: Lawrence Erlbaum Associates, Inc.), 245–263.

[B30] Strömqvist S. Holmqvist K. Johansson V. Karlsson H. Wengelin Å (2006). “What key-logging can reveal about writing,” in *Computer Key-Stroke Logging and Writing: Methods and Applications* eds Sullivan K. Lindgren E. (Amsterdam: Elsevier), 45–72.

[B31] Strömqvist S. Malmsten L. (1998). ScriptLog Pro 1.04 – User's manual. Technical report, Sweden, Gothenburg, Göteborg University, Department of Linguistics.

[B32] Treiman R. Zukowski A. (1988). Units in reading and spelling. J. Mem. Language 27, 466–477 10.1016/0749-596X(88)90068-X

[B33] Van Galen G. P. (1991). Handwriting: issues for a psychomotor theory. Hum. Mov. Sci. 10, 165–191 10.1016/0167-9457(91)90003-G

[B35] Weingarten R. Nottbusch G. Will U. (2004). “Morphemes, syllables, and graphemes in written word production,” in *Multidisciplinary Approaches to Language Production*, ed Pechmann T. Habel C. (Berlin: Mouton de Gruyter), 529–572.

[B36] White S. J. Bertram R. Hyönö J. (2008). Semantic processing of previews within compound words. J. Exp. Psychol. Learn. Mem. Cogn. 34, 988–993. 10.1037/0278-7393.34.4.988 18605883

[B37] Will U. Weingarten R. Nottbusch G. Albes C. (2004). Linguistische Rahmen und Segmentale Informationen bei der Einzelwortschreibung. Evidenzen aus Zeitstrukturen und Fehlerverteilungen. [Linguistic frames and segmental information in writing single words]. Avaliable online at: http://guido-nottbusch.de/doc/Will_Weingarten_Nottbusch_Albes_2002.pdf

[B38] Zwitserlood P. Bölte J. Dohmes P. (2000). Morphological effects on speech production: evidence from picture naming. Langu. Cogn. Processes 15, 563–591 10.1080/01690960050119706

[B39] Zwitserlood P. Bölte J. Dohmes P. (2002). Where and how morphologically complex words interplay with naming pictures. Brain Langu. 81, 358–367. 10.1006/brln.2001.2530 12081405

