# Supplementary material for: Cascaded processing in written compound word production
Source: Front Hum Neurosci. 2015 Apr 21;9:207. doi: 10.3389/fnhum.2015.00207 (PMC4404740; doi:10.3389/fnhum.2015.00207)
Supplement: Supplementary file 1 [file DataSheet1.DOCX]

**Appendix 1**

The 26 target compounds and their translations. The forward slash indicates the constituent boundary.

kilpi/konna ‘turtle’; silitys/lauta ‘ironing board’; linnun/pönttö ‘bird house’; helmi/taulu ‘abacus’; savu/piippu ‘chimney’; tasku/lamppu ‘flashlight’; ranne/kello ‘wrist watch’; moottori/pyörä ‘motorbike’; hammas/harja ‘tooth brush; tuhka/kuppi ‘ashtray’; lohi/käärme ‘dragon’; leivän/paahdin ‘bread toaster’; ruuvi/meisseli ‘screwdriver’; tennis/maila ‘tennis racket; keinu/tuoli ‘rocking chair; sukellus/vene ‘submarine’; toteemi/paalu ‘totem pole’; lento/kone ‘aircraft’; panssari/vaunu ‘tank’; sateen/varjo ‘umbrella’; liikenne/valot ‘traffic lights’; lumi/ukko ‘snowman’; purje/vene ‘sailing boat’; silitys/rauta ‘flat iron’; meri/hevonen ‘seahorse’; pyykki/poika ‘clothespin’

**Appendix 2**

Fixed effects of the final models for each measure in the experiment. *T*-values below -1.96 and above 1.96 correspond to *p*-values below 0.05.

Table A1. WOT: Fixed Effects

|  | Estimate | SE | *t* value |
| --- | --- | --- | --- |
| (Intercept) | 2.2045 | 0.3567 | 6.18 |
| Typical | -0.0690 | 0.0184 | -3.76 |
| LLemfreq | -0.0721 | 0.0158 | -4.56 |
| Syl1Len | 0.0301 | 0.0150 | 2.01 |
| TypingSpeed | 0.3808 | 0.1029 | 3.70 |

Note: WOT values in ms have been log-transformed.

Table B1.1. IKI: Fixed Effects Including All Conditions

|  | Estimate | SE | *t* value |
| --- | --- | --- | --- |
| (Intercept) | 2.7024 | 0.0402 | 67.30 |
| LingTransG | -0.5919 | 0.0824 | -7.18 |
| LingTransM | 0.2330 | 0.0481 | 4.84 |
| LingTransS | -0.0485 | 0.0489 | -0.99 |
| LBiFreq | -0.1109 | 0.0095 | -11.63 |
| LFin3 | -0.0457 | 0.0116 | -3.95 |
| LingTransG:LBiFreq | 0.1602 | 0.0282 | 5.68 |
| LingTransM:LBiFreq | -0.0238 | 0.0191 | -1.25 |
| LingTransS:LBiFreq | 0.0509 | 0.0171 | 2.98 |

Note: IKI values (in ms) have been log-transformed. For LingTrans the NoBoundary (N)

condition has been used as the baseline.

Table B1.2. IKI: Fixed Effect of Bigram Frequency for the NoBoundary Condition (*N*)

|  | Estimate | SE | *t* value |
| --- | --- | --- | --- |
| (Intercept) | 2.1024 | 0.0581 | 68.02 |
| LBiFreq | -0.1060 | 0.0098 | -10.63 |

Table B1.3. IKI: Fixed Effect of Bigram Frequency for the Geminate Condition (*G*)

|  | Estimate | SE | *t* value |
| --- | --- | --- | --- |
| (Intercept) | 2.1024 | 0.0581 | 36.18 |
| LBiFreq | 0.0234 | 0.0193 | 1.21 |

Table B1.4. IKI: Fixed Effect of Bigram Frequency at the Morphosyllabic Boundary (*M*)

|  | Estimate | SE | *t* value |
| --- | --- | --- | --- |
| (Intercept) | 2.8282 | 0.0741 | 38.16 |
| LBiFreq | -0.1274 | 0.0301 | -4.23 |

Table B1.5. IKI: Fixed Effect of Bigram Frequency at Syllable Boundary (*S*)

|  | Estimate | SE | *t* value |
| --- | --- | --- | --- |
| (Intercept) | 2.6174 | 0.0602 | 43.47 |
| LBiFreq | -0.0799 | 0.0200 | -4.00 |

Table C1.1. IKI2: Fixed Effects Including the NoBoundary and Syllable Condition and

Constituent as an Additional Factor

|  | Estimate | SE | *t* value |
| --- | --- | --- | --- |
| (Intercept) | 2.5904 | 0.0508 | 50.95 |
| LingTransS | -0.0649 | 0.0497 | -1.31 |
| LBiFreq | -0.0680 | 0.0127 | -5.36 |
| LFin3 | -0.0476 | 0.0130 | -3.66 |
| Const2 | 0.2130 | 0.0475 | 4.49 |
| LingTransS:LBiFreq | 0.0585 | 0.0174 | 3.37 |
| Const2:LBiFreq | -0.0808 | 0.0162 | -4.97 |

Note: IKI values (in ms) have been log-transformed. For LingTrans the NoBoundary (N)

condition and Constituent 1 have been used as the baseline

Table C1.2. IKI2: Fixed Effect of Bigram Frequency for Constituent 1 of the NoBoundary Condition

|  | Estimate | SE | *t* value |
| --- | --- | --- | --- |
| (Intercept) | 2.5158 | 0.0504 | 49.89 |
| LBiFreq | -0.0742 | 0.0146 | -5.09 |

Table C1.3. IKI2: Fixed Effect of Bigram Frequency for Constituent 2 of the N Boundary Condition

|  | Estimate | SE | *t* value |
| --- | --- | --- | --- |
| (Intercept) | 2.6083 | 0.0515 | 50.68 |
| LBiFreq | -0.1127 | 0.0155 | -7.27 |

Table C1.4. IKI2: Fixed Effect of Bigram Frequency for Constituent 1 at the Syllable Boundary

|  | Estimate | SE | *t* value |
| --- | --- | --- | --- |
| (Intercept) | 2.3766 | 0.1033 | 23.02 |
| LBiFreq | 0.0123 | 0.0368 | 0.34 |

Table C1.5. IKI2: Fixed Effect of Bigram Frequency for Constituent 2 at the Syllable Boundary

|  | Estimate | SE | *t* value |
| --- | --- | --- | --- |
| (Intercept) | 2.7787 | 0.0868 | 32.00 |
| LBiFreq | -0.1421 | 0.0289 | -4.92 |
